# *Rhodiola rosea* Improves Lifespan, Locomotion, and Neurodegeneration in a* Drosophila melanogaster* Model of Huntington's Disease

**DOI:** 10.1155/2018/6726874

**Published:** 2018-06-10

**Authors:** Jasmin G. J. Arabit, Rami Elhaj, Samuel E. Schriner, Evgueni A. Sevrioukov, Mahtab Jafari

**Affiliations:** Department of Pharmaceutical Sciences, University of California, Irvine, Irvine, CA, USA

## Abstract

Huntington's disease (HD) is a dominant, late-onset disease characterized by choreiform movements, cognitive decline, and personality disturbance. It is caused by a polyglutamine repeat expansion in the Huntington's disease gene encoding for the Huntingtin protein (Htt) which functions as a scaffold for selective macroautophagy. Mutant Htt (mHtt) disrupts vesicle trafficking and prevents autophagosome fusion with lysosomes, thus deregulating autophagy in neuronal cells, leading to cell death. Autophagy has been described as a therapeutic target for HD, owing to the key role Htt plays in the cellular process.* Rhodiola rosea*, a plant extract used in traditional medicine in Europe and Asia, has been shown to attenuate aging in the fly and other model species. It has also been shown to inhibit the mTOR pathway and induce autophagy in bladder cancer cell lines. We hypothesized that* R. rosea*, by inducing autophagy, may improve the phenotype of a Huntington's disease model of the fly. Flies expressing HttQ93 which exhibit decreased lifespan, impaired locomotion, and increased neurodegeneration were supplemented with* R. rosea *extract, and assays testing lifespan, locomotion, and pseudopupil degeneration provided quantitative measures of improvement. Based on our observations,* R. rosea* may be further evaluated as a potential therapy for Huntington's disease.

## 1. Introduction

Huntington's disease (HD) is a dominant, late-onset disease characterized by choreiform movements, cognitive decline, and personality disturbance [1]. There is no known cure for HD which affects about 30,000 Americans. It is considered an age-related disease, with the average age of onset between 30 and 50 years [2]. This is caused by a polyglutamine repeat expansion in the HD gene, with expansions greater than 39 glutamine repeats leading to the development of the disease. The mutation of the HD gene, which encodes for the Huntingtin protein (Htt), results in striatal neuron degeneration by causing the dysregulation of autophagic cell processes that facilitate protein clearance [3].

*Drosophila melanogaster*, also known as the fruit fly, is useful for modeling organisms such as humans. Seventy-five percent of human disease genes have a fly ortholog [4], and one of these genes is known to encode for the Huntingtin protein.* Drosophila melanogaster* is considered an optimal model system to study neurodegenerative diseases because of its fully functional nervous system [5]. Fly models of Alzheimer's disease (AD), Parkinson's disease (PD), and spinocerebellar ataxia 3 (SCA3) have been instrumental in the discovery of the molecular basis of these neurodegenerative diseases [6–8]. Alzheimer's disease models of the fly demonstrate adult onset, progressive neurodegeneration, and enhanced mutant tau neurotoxicity [6]. Parkinson's disease fly models which are homozygous for the loss-of-function mutations in the parkin gene were important in discovering the mechanism underlying autosomal recessive juvenile parkinsonism (AR-JP) [7].

Several* Drosophila* models of HD differing in polyglutamine repeat lengths are available to conduct intervention and mechanistic studies. Models varying in the lengths of the polyglutamine repeats have been generated and flies expressing the extended polyQ repeats mimic HD in terms of decreased lifespan, decreased locomotion, and increased photoreceptor degeneration [7–10]. For example, Q75, Q93, and Q120 transgenic flies have polyglutamine lengths of 75, 93, and 120, respectively. It has been established that there is an inverse correlation between the length of the polyQ repeat and the age of onset [9]. The onset of age-related neurodegenerative symptoms in HD is a feature of the HD fly models such as Q75, Q93, and Q120 [10].

The fruit fly has also been extensively studied in aging and anti-aging research, and a number of conserved genes and pathways modulating aging and lifespan have been discovered from these studies [11]. Such genes have been linked to the regulation of metabolic functions, mitochondrial activities, nutrient sensing, and protein synthesis [12, 13]. Since the molecular events surrounding aging and longevity have been well-characterized in the fly, this model can potentially be used to study interventions that delay the progression of age-related diseases in fly models that display age-related phenotypes such as the HD fly model.

One possible approach to alleviating the symptoms of HD is to use the root extract of* Rhodiola rosea. *This plant has been used in traditional and integrative medical practices in Europe and Asia where it is prescribed to improve mood and physical and mental stamina and to enhance protection against high altitude sickness [14]. The putative active compounds of* R. rosea*, rosavins and salidroside, are used to characterize the extract. The extract has also been shown to attenuate aging in* C. elegans, D. melanogaster, and S. cerevisiae.* In the case of* D. melanogaster*, the extract increased lifespan up to 24% through a mechanism independent of dietary restriction, a well-established intervention to increase lifespan and healthspan of various organisms [15, 16].* Rhodiola rosea* and its putative active compound, salidroside, has been shown to inhibit the mTOR pathway and induce autophagy in bladder cancer cell lines [17]. Since the lifespan extension properties of* R. rosea* appear to be conserved among various species,* D. melanogaster*,* C. elegans*, and* S. cerevisiae *[18, 19], this plant may be a viable intervention to attenuate the symptoms of age-related diseases in humans such as HD.

## 2. Materials and Methods

### 2.1. *Rhodiola rosea* Extract

The* R. rosea* extract used for this study, SHR-5, was obtained from the Swedish Herbal Institute. It contains 3.5% rosavins and 1.4% salidroside (HPLC data on file).

### 2.2. *Drosophila melanogaster* Stocks

The polyQ-expressing transgenic line, 4F1, was a gift from J. Lawrence Marsh from UC Irvine. This line, containing 93 polyglutamine repeats in exon 1 of the Huntington gene, will be referred to as UAS-HttQ93 [20, 21]. The Gal4 driver used was the pan-neuronal elav driver, elavC155 from the Bloomington Drosophila Stock Center at Indiana University. Male elav-Gal4 were mated with female UAS-HttQ93 to produce offspring with females expressing the extended polyQ repeat (elav-Gal4>UAS-HttQ93) in all nerve cells. These flies will be referred to as “HD flies.” The expression of the repeats was lethal to males in the late larval stage, so only females were used for assays performed on adult flies. Assays using larvae required a cross between female elav-Gal4 and male UAS-HttQ93, resulting in all offspring expressing the 93 polyQ repeats.

### 2.3. Larval Feeding

Two days after HD flies laid eggs (Day 0), eggs (elav-Gal4>UAS-HttQ93) were transferred into food with* R. rosea* extract mixed in. To make the* R. rosea* food, the banana food was cooked as detailed in Schriner et al. [15]. After cooling the food to 48°C, 500 mL of the food was transferred into a blender along with 12.5 g of* R. rosea* extract (500 mL x 25 mg/mL). The mixture was blended until homogenous, then a pipet-aid w/a 10 mL serological pipet was used to measure out 5.5 mL of the* R. rosea* food into vials. The fly eggs were reared in this food until they reached the pupal stage.

### 2.4. Adult Feeding

As described in Schriner et al. [15, 22], a 3% yeast solution with 25 mg/mL of* R. rosea* extract was made, and 75 *μ*L of the solution was added into each vial and allowed to dry to create a layer of yeast. Flies were transferred to new vials with food every other day.

### 2.5. Feeding Treatments

The four treatment regimens are summarized in Table 1.

### 2.6. Lifespan

Lifespan assays were performed as described in Schriner et al. [15]. 40 vials per group with 12 female HD flies in each vial were set up. Flies were transferred to new food every other day, with the deaths counted on transfer days.

### 2.7. Rapid Iterative Negative Geotaxis (RING) Assay

Sixty vials with 12 female HD flies in each were set up for this assay. Flies were transferred to new food every other day. To perform the RING assay, the 12 flies from each feeding treatment were transferred into empty vials and loaded onto the RING assay apparatus [23]. Six trials were done, using a total of 72 flies per group. As a video of the setup was being taken, three rapid strikes were applied to the apparatus. Video recording was stopped after 6 seconds. Using ImageJ, the climbing heights of the flies were measured 4 seconds after the last tap.

### 2.8. Pseudopupil Assay

Sixty vials with 12 female HD flies in each were set up for this assay. After 7 days of feeding with* R. rosea*, fly heads were decapitated and mounted on a slide using clear nail polish and were observed using a Zeiss Scope.A1 microscope with an N-ACHROPLAN 100x/1,25 oil lens. Eight eyes from eight different flies were randomly selected from each feeding treatment. A minimum of 50 ommatidia were counted per eye, with the observer blinded to the identity of each group. Rhabdomeres were counted based on visibility, not on shape, size, or brightness [21, 24].

### 2.9. Larval Crawling Assay

Two days after eggs were laid, eggs were transferred into food with* R. rosea *(Day 0). On Day 5, larvae were collected by adding a 20% sucrose solution into the vial and waiting 20 minutes until the larvae floated to the top. Ten larvae from each group were collected using a 200 *μ*L pipet with the tip cut off and loaded onto the center of the plate with 2% agarose placed on top of graphing paper. The larvae were allowed to crawl from the center of the plate for one minute, after which a picture of the plate was taken [25]. Crawling distances were measured using ImageJ.

### 2.10. Eclosion Assay

Ten vials containing 25 mg/mL of* R. rosea* and 10 vials with control food were set up with 5 males and 5 females in each vial. The vials were left in the incubator at 25°C for 24 hours. After 24 hours, the vials were cleared of flies and eggs were left in the vials. On Day 11, the number of eclosed flies were counted every 3 hours [25].

### 2.11. Statistical Analysis

Data analysis for all the assays was performed using GraphPad Prism 7. For the lifespan assays, Log-Rank (Mantel-Cox) Test and Tukey's Multiple Comparison Test were used. Mean increases in lifespan were analyzed using ANOVA multiple comparisons test. This test was also used for the RING, pseudopupil, larval crawling, and pseudopupil assay.

## 3. Results

### 3.1. *Rhodiola rosea* Extends Lifespan of Adult-Fed Female HD, elav-Gal4, and UAS-HttQ93 Flies

**Figure 1:**
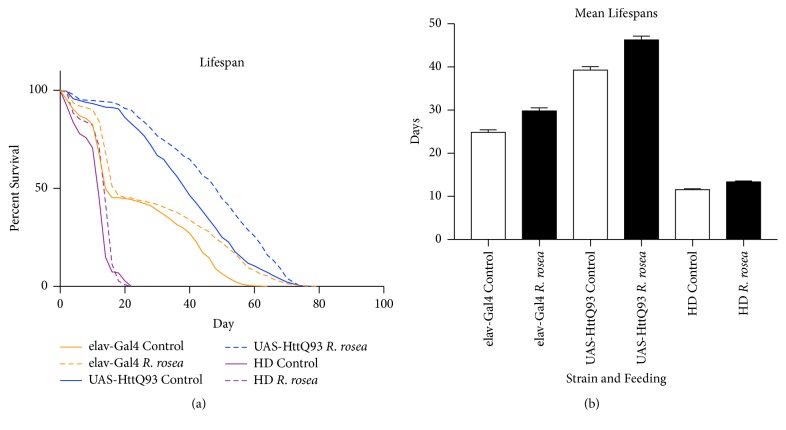
The effect of* R. rosea* on female* D. melanogaster* lifespan. (a) Compared to control-fed flies, HD, elav-Gal4, and UAS-HttQ93 flies showed a mean lifespan increase of 25%, 21%, and 17%, respectively. n=480, p<0.0001 for each strain, Log-Rank (Mantel-Cox) Test. (b) Analysis of the mean lifespan increases shows significant differences between the two treatments for elav-Gal4 and UAS-HttQ93 but not for HD flies. *∗∗∗∗*p<0.0001, ANOVA multiple comparisons test.

A lifespan assay was performed to measure the effects of the extract on the mean lifespan of the short-lived HD flies and control flies to observe if the effects were conserved among different fly strains. It has already been observed that flies expressing 93 polyQ repeats have reduced lifespan [26], but the lifespan differences between these flies and the parents used to make the cross were yet to be studied.* Rhodiola rosea* increased the mean lifespan of the parent strains, elav-Gal4 and UAS-HttQ93, and the HD flies by 25%, 21%, and 17%, respectively. The increase in lifespan was found to be significant for each strain (Figure 1).

### 3.2. *Rhodiola rosea* Extends the Lifespan of Larvae-Fed Female HD Flies

**Figure 2:**
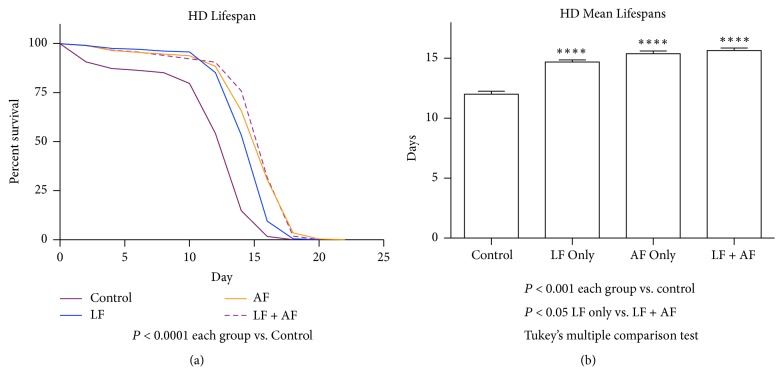
The effect of* R. rosea* on the lifespan of HD flies. Relative to HD control flies (a) HD LF, AF, and LF + AF flies exhibited the following lifespan increases: 22%, 28%, and 31%, respectively. P<0.0001 (each group versus control). (b) All three groups supplements with* R. rosea* resulted in increased mean lifespan. P<0.001 (each group versus control); P<0.05 (LF only versus LF+AF), Tukey's multiple comparison test. n=240.

Figure 1 shows that* R. rosea* improves lifespan of HD flies when fed as adults, but the effect of the extract on fly lifespan when fed to the HD flies as larvae was yet unexplored. Lifespan assays were performed to observe the impact of feeding* R. rosea* during larval and adult stages of HD flies. This was done to elucidate the impact of the extract on lifespan when fed to the flies at varying stages in life. All feeding treatments increased the mean lifespan with feeding during both larval and adult stages having the largest increase in lifespan (Figure 2). The differences between the feedings of* R. rosea*, however, were less pronounced. The only significant difference we found was between the lifespans of the flies fed as larvae and those fed as both adult and larvae.

### 3.3. *Rhodiola rosea* Improves the Locomotion of Larvae-Fed Adult HD, elav-Gal4, and UAS-HttQ93 Flies

**Figure 3:**
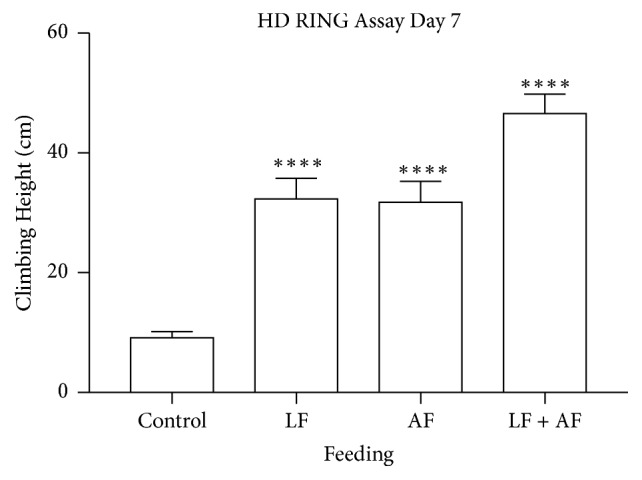
The effect of* R. rosea* on the climbing heights of adult HD flies. Flies fed* R. rosea* displayed significantly increased climbing heights compared to the control group. n~75, *∗∗∗∗*p<0.0001, ANOVA multiple comparisons test.

To observe the effects of the extract on the locomotion of the HD flies, a RING (Rapid Iterative Negative Geotaxis) assay was performed. This assay exploits the innate response of* Drosophila* to escape by ascending up the walls of a vial after being tapped to the bottom of the vial [23]. This response decreases as flies age so the RING assay can be used to evaluate the impact of interventions such as* R. rosea* on age-related decline in locomotion.* Rhodiola rosea* increased the mean climbing heights of adult HD flies when supplemented during the larval stage, as adults, or both. Of note, when* R. rosea* was supplemented to both larva and adults, the positive effect was more significant (Figure 3).

### 3.4. *Rhodiola rosea* Increases the Mean Rhabdomere Count in HD Flies

**Figure 4:**
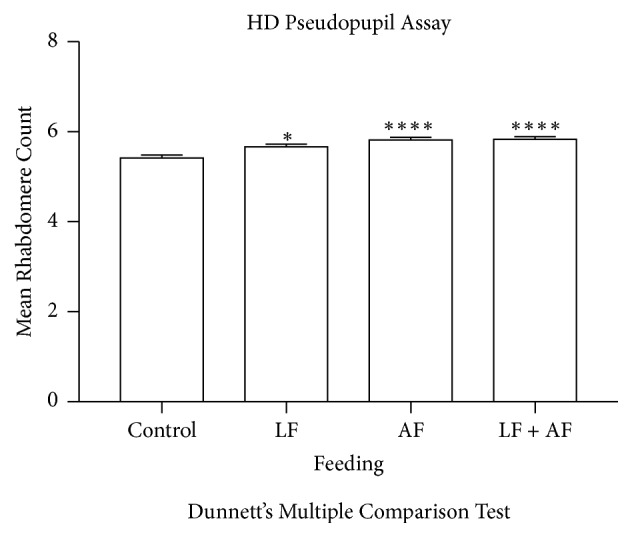
The effect of* R. rosea* on the neurodegeneration of HD fly rhabdomeres. HD flies fed* R. rosea* exhibited significant rhabdomere count increases compared to control. Data analysis was performed using an analysis of variance (ANOVA) *∗*p<0.05, *∗∗∗∗*p<0.0001, n=50.

The HD flies used in this experiment had 93 polyglutamine repeats and demonstrated neurodegeneration which can be observed by counting rhabdomeres within the compound eye. The pseudopupil assay is fast and sensitive, which allows for the quantification of the degree of neurodegeneration in an* in vivo* model [21]. The supplementation of* R. rosea* at any age, larvae and adult, increased the mean rhabdomere count in the HD flies (Figure 4).

### 3.5. *Rhodiola rosea* Attenuates the Crawling Distance of HD and UAS-HttQ93 Larvae

**Figure 5:**
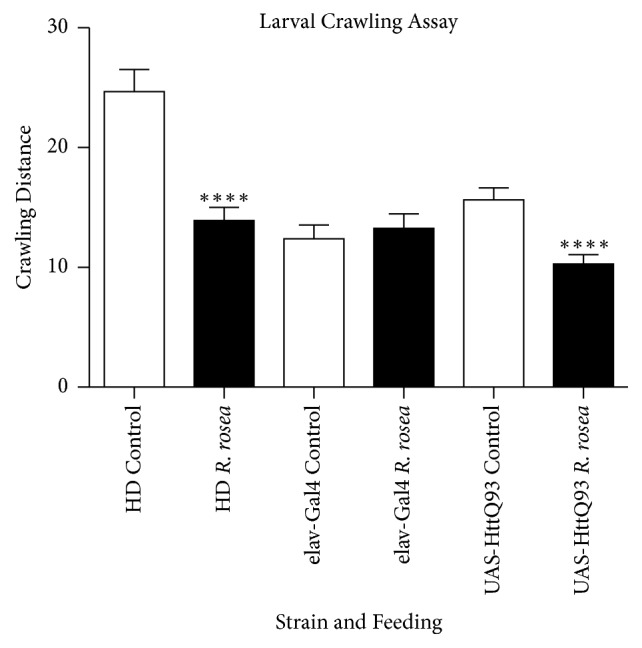
The effect of* R. rosea* on the crawling distances of larvae. Significant decreases in larvae crawling distances were observed in HD and the UAS-HttQ93 strains that were fed* R. rosea. *n =10, ANOVA multiple comparisons test.

The larval crawling assays were performed to observe the possible effects of* R. rosea* on HD fly development. This assay is a developmental assay used to study the possible toxic effects of compounds on larvae locomotion [25]. Larvae studies were performed on HD flies and control flies to observe if the effects of the extract were conserved among the strains.* Rhodiola rosea* induced a decline in mean larval crawling distance in HD and UAS-HttQ93 flies (Figure 5).

### 3.6. *Rhodiola rosea* Decreases the Percent Eclosion and Eclosion Rate of HD, elav-Gal4, and UAS-HttQ93 Flies

**Figure 6:**
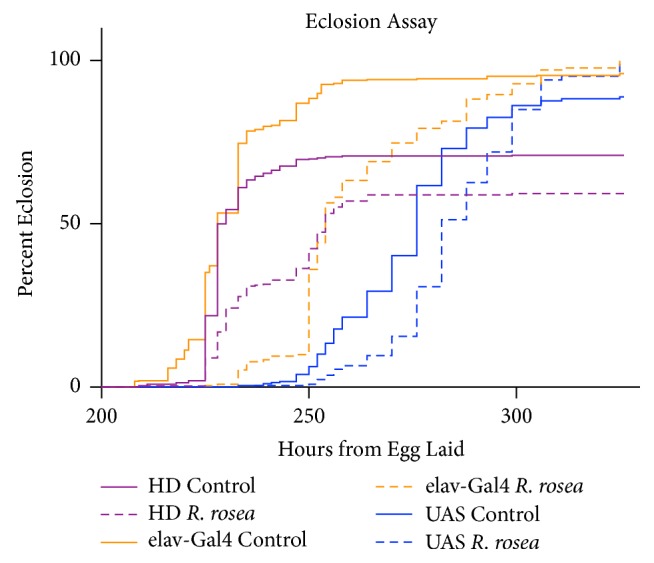
The effect of* R. rosea* on eclosion rates and percent eclosion. HD and UAS-HttQ93 flies demonstrated decreases in the percentage of flies transitioning from the pupal stage to the adult stage. HD, elav-Gal4, and UAS-HttQ93 flies showed significant decreases in eclosion rates by 5%, 13%, and 5%, respectively. The p values for the eclosion rate decreases were all significant (p<0.0001). Log-Rank (Mantel-Cox) Test.

Another developmental assay used to observe the effects of* R. rosea *on the HD larvae was the eclosion assay which is used to measure the effect of toxins on* Drosophila* throughout the larvae-pupal stage [27]. The number of flies emerging from the pupal stage was counted to show the effects of* R. rosea* on the eclosion rate and percentage of the emerged adult flies.* Rhodiola rosea *decreased the percentage of flies transitioning from the pupal stage to the adult stage and the eclosion rates of HD, elav-Gal4, and UAS-HttQ93 flies (Figure 6).

## 4. Discussion

Huntington's disease (HD) is a dominant, late-onset disease characterized by choreiform movements, cognitive decline, and personality disturbances [1]. Since it is considered an age-related disease, treatments that slow aging may slow the progression of HD or alleviate its symptoms. One such potential treatment may be the root extract of* Rhodiola rosea*. In this work, we evaluated the impact of* R. rosea* on a fly model of HD. We found that* R. rosea* could prevent neurodegeneration, improve locomotion, and increase lifespan. Given that the positive impacts of* R. rosea* on lifespan appear to be conserved among various species such as* S. cerevisiae, C. elegans, *and* D. melanogaster *[18, 19] and that the plant has demonstrated many therapeutic effects in clinical studies [28–31], this extract may be a viable treatment for symptoms associated with HD in humans.

The Huntingtin (Htt) protein has been implicated in the regulation of selective macroautophagy, particularly in its role in vesicle trafficking and autophagosome formation. The mutant form of the protein, mHtt, impairs the retrograde transport of vesicles to the neuron cell body, cargo loading, and the fusion of autophagosomes and lysosomes, thus leading to an abundance of empty autophagosomes and an accumulation of toxic materials in the cytoplasm [3]. Fly models of Huntington's disease (HD) have shown downregulated levels of autophagy due to the inability of the cell to degrade damaged organelles or aggregated proteins [3]. The mutant Huntingtin protein (mHtt) has also been implicated in caspase activation leading to increased toxicity and cell death [32]. The downregulation of autophagy in animal models has been linked to neurodegeneration [33], thus positing autophagy as a therapeutic target for diseases such as Parkinson's disease, Alzheimer's disease, and Huntington's disease [34].

It appears that alleviating the symptoms and pathology of HD involves increasing the rate of autophagy. Researchers have observed improvements in HD disease phenotypes in fly and mouse models using mTOR inhibitors [34] and HDAC inhibitors [35]. Rapamycin, an inhibitor of mTOR, was shown to prevent the accumulation of mutant Htt (mHtt) proteins leading to cell death in cell models of HD and also conferred neuroprotective effects in a fly model [36]. Although rapamycin was found to be effective in inducing autophagy and delaying symptoms of aging in various animal models [37], adverse effects such as immunosuppression and glucose intolerance have been observed when the compound was used in humans [38], making it unsuitable for long-term prophylactic use.

Here we suggest that a standardized root extract of* Rhodiola rosea* (SHR-5) may be considered as a possible therapy to alleviate the symptoms of HD in humans. By supplementing an HD fly model with* R. rosea*, we observed improvements in the HD phenotype in terms of lifespan, locomotion, and neurodegeneration. The extract has also been shown to inhibit the mTOR pathway and induce autophagy in bladder cancer cell lines [19]. Although* R. rosea* was shown to act independently of the mTOR pathway in the fly model [15], the effects of the extract on rates of autophagy of* D. melanogaster* have not yet been observed. Currently, there are no reported adverse effects or drug interactions for* R. rosea* [29–31], making it a promising preventive treatment for Huntington's disease in humans.

The HD fly model that was used in this work expresses 93 polyQ repeats. A key characteristic of these HD flies is the rapid degeneration of the compound eye. By visually analyzing the rhabdomeres contained within the ommatidia of the fly eye, photoreceptor loss was quantitatively measured. The HD flies demonstrated decreased photoreceptor loss when supplemented with* R. rosea* as larvae only, adults only, and both adults and larvae. This suggests that the effects of the plant extract on neuronal loss were not dependent on the life stage and that feeding during only the larval or adult stage yields equally positive results as feeding during both life stages. Although the anti-aging mechanism of* R. rosea* is not known, we and others have found that* R. rosea* can induce autophagy [17, 33, 39]. Thus, one possibility is that* R. rosea*, through the induction of autophagy, can directly counteract the negative effects of the polyQ repeats. While being certainly an attractive hypothesis, it seems unlikely because of the marginal effects of* R. rosea* on the rhabdomere number relative to the marked effects that it has on locomotion. A more plausible explanation could be an indirect effect of* R. rosea. *Interestingly,* R. rosea* still resulted in a positive effect in adults when fed to larvae only. This suggests that the mechanism of action of* R. rosea* could be related to alteration in gene expression.

Adult HD flies also display impaired locomotion in the form of decreased climbing heights compared to the parental strains. We found that* R. rosea* increased locomotion in adult HD flies. However, surprisingly, the extract improved locomotion in the adult stage to equivalent degrees whether fed during adult or larval stages. The effect on locomotion during the adult stage was even greater when fed to both. The magnitude of effect and the benefit of larval feeding are both striking. Again, we can only speculate on the mechanism, but it appears that feeding* R. rosea* during either development or adult stages resulted in improved locomotion. Since the polyQ repeats in the HD fly model are expressed during the larval stages [21], we can speculate that* R. rosea* prevents polyQ repeats from aggregating before a significant problem can occur. If this were the case, why then is the effect on rhabdomere formation relatively marginal when the extract is fed to larvae? It may also be that* R. rosea* protects different cell lineages to differing degrees considering that the extract protected motor neurons more significantly than rhabdomeres. In such a case,* R. rosea* may have a more positive impact on physical performance in humans with HD, as suggested by its positive impact on locomotion in HD flies, as opposed to a marginal positive impact on the pathology of HD in humans, as suggested by the effects seen in the rhabdomere assay in HD flies.

With respect to adult lifespan, we observed that* R. rosea* had a positive effect in all of the three fly strains used in this study, the disease model, and the two parental controls. This suggests that, with respect to lifespan alone,* R. rosea* may not directly be benefiting only HD flies. It is more likely that* R. rosea* confers an overall strain independent increase in lifespan. This may not necessarily be an issue, as the extract did provide a modest decrease in pathology, but a marked increase in physical performance. Thus, an improved lifespan coupled with improved health can only be seen as a benefit. As for the locomotion assay,* R. rosea* increased lifespan whether fed to adults, larvae, or both. This clearly demonstrates that* R. rosea* mediates changes during development which affect the adult animal. The effect on lifespan was somewhat different from what was seen for locomotion in that there appeared not to be an additive effect between larval and adult feeding. Thus, for lifespan it did not seem to matter when* R. rosea* was added; the effect is the same.

To evaluate whether* R. rosea* had any harmful effects on the larvae of HD flies, we examined its impact on the percent eclosion, rate of Eclosion, and larval crawling. In all these cases,* R. rosea* was found to have a negative effect; it slowed larval crawling assay, decreased percent Eclosion, and delayed the time of eclosion. These types of assays typically indicate a toxic effect of a given agent. It may be that the extract slowed development, giving the larval cells additional time to clear the polyQ repeats before they can cause much damage. The larvae then moved on to the next stages of development possessing lesser amounts of the polyQ repeats. Such clearance may have conferred improved locomotor ability and longer lifespan. The negative effects of the extract on the larvae may have also been due to a nonoptimal dose. Further studies are warranted.

In summary*, R. rosea* seemed to provide beneficial effects in a fly model of Huntington's disease. These beneficial effects were observed when* R. rosea* was supplemented to either larvae alone, adults alone, or both. While the mechanism is not clear, it may be that* R. rosea* activates autophagy which counteracts the negative effects of the mutant Huntingtin proteins. Despite not knowing how* R. rosea* might work, due to its positive effects on other organisms and its overall excellent safety profile,* R. rosea* may be further studied as a potential new and natural therapy to alleviate the symptoms of Huntington's disease.

## 5. Conclusion

In conclusion, our results show that the supplementation of* R. rosea* to the Q93 adult model of Huntington's disease improves its phenotype in terms of lifespan, locomotion, and neurodegeneration. Although the extract appeared to have a toxic effect on the larval crawling and eclosion rate, it still seemed to have a positive impact on the phenotype of adults that were fed as larvae. More studies need to be performed to evaluate the mechanism of action of the toxicity observed during the larval stage. In summary,* R. rosea* extract may be further studied as a possible therapy to alleviate the phenotype and symptoms of Huntington's disease.

## Figures and Tables

**Table 1:** The four treatment regimens used in this study.

**Group**	**Feeding Treatment**
Control	0 mg/mL *R. rosea* as larvae + 0 mg/mL *R. rosea* as adults

Larvae Feeding (LF)	25 mg/mL *R. rosea* as larvae + 0 mg/mL *R. rosea* as adults

Adult Feeding (AF)	0 mg/mL *R. rosea* as larvae + 25 mg/mL *R. rosea* as adults

Larvae Feeding and Adult Feeding (LF + AF)	25 mg/mL *R. rosea* as larvae + 25 mg/mL *R. rosea* as adults

## Data Availability

The data used to support the findings of this study are available from the corresponding author upon request.
